# Spectroscopic Detection of Biosignatures in Natural Ice Samples as a Proxy for Icy Moons

**DOI:** 10.3390/life13020478

**Published:** 2023-02-09

**Authors:** Francisco Calapez, Rodrigo Dias, Rute Cesário, Diogo Gonçalves, Bruno Pedras, João Canário, Zita Martins

**Affiliations:** 1 Centro de Química Estrutural, Institute of Molecular Sciences and Department of Chemical Engineering, Instituto Superior Técnico, Universidade de Lisboa, 1049-001 Lisboa, Portugal; 2 Institute for Bioengineering and Biosciences, Instituto Superior Técnico, Universidade de Lisboa, 1049-001 Lisboa, Portugal; 3 Associate Laboratory i4HB—Institute for Health and Bioeconomy, Instituto Superior Técnico, Universidade de Lisboa, 1049-001 Lisboa, Portugal

**Keywords:** Europa, Enceladus, planetary analogues, astrobiology, spectroscopy

## Abstract

Some of the icy moons of the solar system with a subsurface ocean, such as Europa and Enceladus, are the targets of future space missions that search for potential extraterrestrial life forms. While the ice shells that envelop these moons have been studied by several spacecrafts, the oceans beneath them remain unreachable. To better constrain the habitability conditions of these moons, we must understand the interactions between their frozen crusts, liquid layers, and silicate mantles. To that end, astrobiologists rely on planetary field analogues, for which the polar regions of Earth have proven to be great candidates. This review shows how spectroscopy is a powerful tool in space missions to detect potential biosignatures, in particular on the aforementioned moons, and how the polar regions of the Earth are being used as planetary field analogues for these extra-terrestrial environments.

## 1. Introduction

Ice is ubiquitous in the outer solar system as the latter is populated by several bodies either composed of or covered in ice, such as comets [1,2], “primitive” asteroids [3], Kuiper belt objects (KBOs) [4], and some of the satellites of Jupiter and Saturn [5,6]. Data from space missions show water ice on the surface of comet 67P/Churyumov–Gerasimenko detected by the Rosetta visible and infrared thermal imaging spectrometer (VIRTIS), and imagery by the New Horizons spacecraft suggested cryovolcanoes on Pluto [7,8]. Water ice is the predominant ice type in outer solar system bodies (such as in the icy Galilean and Saturnian moons), while other volatile species (e.g., CO_2_, NH_3_, CH_4_, and SO_2_) may be present as minor components of the ice [9,10,11,12]. In some KBOs, such as Pluto, nitrogen ice is the main surface component, with traces of CH_4_ and CO [13]. Some of the icy Galilean and Saturnian moons may be oceanic worlds, with significant amounts of liquid water locked between an icy crust and a silicate core, as observed by the Galileo and Cassini missions in Europa and Enceladus, respectively [14,15]. These icy moons with a subsurface ocean are also the ideal place to evaluate how extra-terrestrial water oceans may harbour and sustain life [16].

Three essential characteristics must be met for a location to be deemed habitable: (1) proper physicochemical conditions (such as a solvent); (2) a source of energy (tidal heat, solar radiation, radioactive decay, or geochemical energy); and (3) the essential elements that all known life requires (carbon, hydrogen, nitrogen, oxygen, phosphorus, and sulphur, CHNOPS) [16]. The presence of these characteristics on planetary bodies can also be more broadly categorized as a first-order evaluation of whether a planetary body is likely to host habitable conditions on some portion of its surface or interior [16,17]. These planet-specific conditions of habitability have been found on, for instance, Earth, Mars, Europa, and Enceladus [18].

Focusing on the possibility of life in the liquid water layers of ocean worlds, the material exchange with the icy surface may not only support the habitability of these environments, but also bring to the surface signatures of putative living phenomena taking place deep below [19], especially in chaotic terrains [20]. Indeed, Europa and Enceladus’ dynamic characteristics and relatively youthful surfaces offer strong proof that their oceans are constantly interacting with their surrounding frozen capsule [19]. This is crucial validation for the importance of characterizing the icy surface of these worlds [19]. To this end, spectroscopic techniques offer advantages such as the ability to perform remote analyses and the possibility to support the data with validated spectra libraries, overall enabling accurate assignments of specific functional groups, and higher resolution in very cold environments, where there is less broadening of the peaks [21].

Limited by the absence of a fast, easy, and affordable accessibility to the surface of ocean worlds, astrobiologists frequently study planetary field analogue sites on Earth that mimic one or more conditions from such planetary bodies [22]. In particular, cold and dry environments such as those found in the polar regions of Earth, are ideal to study characteristics of the surface of the icy moons of Jupiter and Saturn [22]. From the biology of the extremophiles to the freezing dynamics of waters, contamination control strategies/protocols [23,24,25], and instruments and rovers, the (Canadian) Arctic and several sites of Antarctica are being used to test methodologies and protocols, and to train scientists and machines to better understand the extreme environments presented by Europa and Enceladus [26].

In this review, we summarize the state-of-the-art chemical mapping of Europa and Enceladus through spectroscopic methods with the aim of assessing possible biosignatures. We also review how planetary field analogues complement the data provided by space missions to accurately assess the chemical dynamics of the icy moons of Jupiter and Saturn.

## 2. Habitability on Icy Moons

### 2.1. The Icy Moons of Jupiter

Jupiter, the fifth planet of the solar system, harbours dozens of moons [27], among them, some icy moons, including Calisto, Ganymede, and Europa that garner most of the focus from the scientific community in terms of the potential of habitability [28].

**Figure 1:**
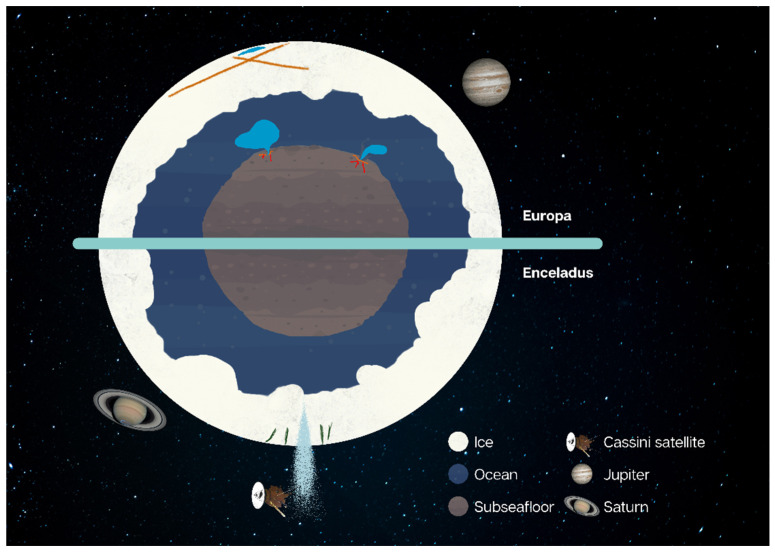
(Top half) Composition of Europa, highlighting the icy crust with its characteristic ridges atop a liquid ocean. The plumes of Europa are not depicted, as these are small and more difficult to identify. (Bottom half) Structure of Enceladus with its plumes ejecting material from the tiger stripes in the South Pole. (Note that Europa and Enceladus are not to scale, as Europa is more than six times the diameter of Enceladus.) Depiction of the Cassini spacecraft performing a flyby. Image credit to Catarina Miranda. Images of Cassini, Saturn and Jupiter presented are credited to ESA and NASA [34,35,36].

The surface of Calisto is made of ancient water ice and has sustained impacts for billions of years [29]. Ganymede, on the other hand, holds evidence all over its surface of dynamic geological processes of especially early internal activity, expressed as grooves and ridges [29]. Such evidence reaches us from pictures taken by the Galileo spacecraft [30]. Europa has an extremely fractioned surface due to subsurface activity, with very few impact craters, indicating a young outer shell [31], renovated because of its subsurface ocean which is maintained in the liquid phase by the internal tidal heat, presence of salts, radioactive decay, or geochemical energy (from water-rock interactions) [32]. Measurements of Europa’s induced magnetic field, and considering the thickness of its rocky layers, point to a high concentration of ions in its ocean, theorized to have been leached in the differentiation process [33]. In addition, the moon’s moment of inertia supports the hypothesis of a hydrosphere atop the lithosphere, together with an iron core in the centre [29]. Figure 1 depicts such structure.

Even with high water content, conditions for life are neither present on Ganymede nor Calisto [37]. Ganymede, the largest moon of Jupiter, is thought to hold more water than Europa, for example [37]. Nevertheless, the dynamics in place rule out this massive moon as a candidate in the search for life [38]. Recent models point to several layers of ice sheets intercalating with water due to the heat flow and high-pressure Ganymede experiences [38]. These icy barriers would block possible gradients of material relevant for biology, from the silicate mantle to the ocean where life would be feasible to exist [38]. The work published by Journaux et al. [37] shows that it is possible for some salts and inorganic material from the silicate mantle to become partitioned into this high-pressure ice, and be released into the ocean; a thought-provoking mechanism for a flux of material into Ganymede’s water ocean [37]. Such high-pressure conditions are not present in the boundary between the silicate mantle and the water ocean of Europa, allowing for direct geological interaction between these two [39]. This may also allow for hydrothermal activity like that occurring on Earth, known for providing the chemical disequilibria able to sustain biological primary production in the absence of sunlight [40].

Harsh conditions on the top layer result from the strong magnetic field of Jupiter capturing and accelerating charged particles and bombarding them into Europa with electrons and ions from Io’s plasma torus, in a pattern of deposition predominantly at the apex of the trailing hemisphere [41]. Such energized bombardment can also dissociate the H_2_O molecule into H_2_ gas and reactive oxygen [42]. The extremely oxidizing environment found on the surface, acts upon molecules that may eventually make their way back to the ocean through gradual resurfacing [43], and later possibly through subduction (surface area removal) by salt content difference producing density difference [44].

Regarding energy as one of the pillars for habitability, not only might Europa have a similar hydrothermal system as is found on Earth, but if charged particles find their way into the ocean from the surface, it might deliver necessary elements to fuel the chemical imbalance needed for life [45]. This adds to the importance of better understanding the circulation of matter in the ice shell of Europa, and the dynamics it has with the ocean with which it is in contact [45]. Furthermore, the existence of a global ocean that separates Europa’s icy exterior from its rocky interior allows a direct interaction between the liquid water and the lithosphere [45]. Reactions between water and rock could provide the ocean with electron donors, while surface irradiation could generate oxidants (such as H_2_SO_4_), which could then endow the water ocean with electron acceptors [45]. Just as such redox disequilibria are exploited by chemoautotrophs on Earth, they could potentially feed living phenomena in the ocean of Europa [45]. It must be noted that chemical reactions occurring on the surface of icy bodies in the outer solar system are driven by UV photons (up to less than one centimetre depth), ionizing radiation (as deep as a metre or two below the surface), and thermal processes [33].

Europa’s surface seems to be crisscrossed with fractures that interlink with each other in a complex history of episodes [46,47]. The bands created from proto-tectonic processes are also frequently found where the surface suffers events similar to divergent faults (as found on Earth), where the ocean being forced upwards pulls apart the icy layer on top [46]. In addition to these fractures, surface images show other interesting surface features like chaotic terrains, thought to be created by liquid processes when a plume rises [46,48,49]. Ultimately, there are several mechanisms acting on Europa that deliver oceanic material to the surface of the moon that may justify surface investigations via remote sensing [50].

Laboratory models of water-rock interactions using analogue molecules predict high concentrations of magnesium and sulphate salts in Europa’s ocean [51]. Both Galileo’s near-infrared mapping spectrometer and Cassini spacecraft analysis have confirmed these models [21]. The Keck Observatory in Hawaii equipped with adaptive optics system and the near-infrared integral field spectrograph called OSIRIS together with the Very Large Telescope in Chile, have a much larger spectral resolution (~40 times higher) than the instruments onboard the Galileo spacecraft [21]. These technologies added another piece of information when mapping the surface of Europa and showed sulphates on the entire global surface [21]. While these images point to a high abundance of sulphates on the bands and chaotic regions, they mainly show, on a global scale, a peak on one hemisphere of Europa [39]. To understand why they are concentrated in one hemisphere, we must consider the orbital mechanics around Jupiter: Europa, similarly to the other Galilean moons, is tidally locked, i.e., the same face of the moon is constantly facing Jupiter [46]. This means that while orbiting Jupiter, Europa has a leading hemisphere facing the direction of travel and a trailing hemisphere facing away from it [46]. Based in these differences between hemispheres, current theories suggest that the concentration of sulphates on the trailing hemisphere, might arrive from an exogenous source, possibly Io’s ejected material from its high volcanic activity [46,52].

The Hubble Space Telescope studies of Europa’s surface revealed a spectral pattern that was closely associated with endogenous characteristics and was compatible with irradiated sodium chloride [50]. The electrons, being incorporated into the halogen vacancies in the crystal lattice of the salt, can emit light at a different wavelength creating a coloured centre [53]. This renders the salt with a visible change of colour to a similar appearance as that seen on those geological features in the icy shell [53]. Connecting those coloured ridges with radiated surfaced sodium salts, leads to the hypothesis that the ocean from which they originated is also rich in this element [19]. If the NaCl salts found atop have in fact their genesis in the ocean and were later brought to the surface by geological processes, a chlorine rich ocean is expected [53]. Alternatively, the ocean may be rich in sulphates that were lost in the formation of the ice shell and the surface is not a direct representation of the ocean chemistry. Recent studies by Fox-Powell [45] on the freezing process of water with a complex mixture of salts, show that sulphates precipitate with a decrease in temperature and the freezing of water [54]. The ocean water of Europa is warmer than the ice shell so, during the drop in temperature, when approaching the freezing point, the sulphates would have precipitated, leaving the fluid enriched on the other salts, namely chlorine [45].

### 2.2. The Icy Moons of Saturn

The Saturnian system has many natural satellites, several of them icy moons [6]. Here, we will discuss Saturn’s icy satellites using data from the highly successful NASA-ESA Cassini-Huygens mission, (henceforth shortened to Cassini). The Cassini spacecraft orbited Saturn’s system (thus being an orbiter as opposed to Huygens which was a lander) from mid-2004 until 15 September 2014, when it was commanded to crash into Saturn to protect the ocean world of Enceladus from its contamination [55]. The significance of the ice satellites lies in their ability to help us better comprehend the geological diversity of the several moons and how they interact within the intricate Saturnian system [55].

When it comes to icy satellites, Enceladus has been the standout moon, but Cassini also found several other unexpected findings at the other frozen satellites. These include Iapetus’ equatorial ridge and Hyperion’s sponge-like look [56,57], the bright ray system on Rhea due to a recent impact, and thin streaks on Dione with a tectonic origin [58,59]. The dark material on Iapetus, which appears to be just a thin surface coating, is made up of organic materials [60]. Several of the material’s spectral characteristics match those of other moons in Saturn’s system, including Phoebe, Hyperion, Dione, and Epimetheus as well as the F ring and Cassini division [53]. It has been verified that the dark material for Phoebe appears to have originated outside the Saturnian system [61]. All of the satellites’ abundance of impact craters were confirmed by Cassini’s increased resolution and worldwide mapping coverage, which also showed that Tethys, Rhea, and Dione’s endogenic activity varied to differing degrees [52].

In regard to habitability, Enceladus is considered a promising site for investigation and special focus is deserved. This moon is the main source of material for Saturn’s E ring, due to the 100 km sized plumes emanating from its south pole, ejecting water ice and water vapour [54,62]. This is released from aligned vent structures, named tiger stripes, that are fed by a subsurface ocean that experiences ongoing hydrothermal interaction with a silicate core as shown in Figure 1 [63]. Grains found in the E ring sourced from Enceladus contain sodium salts amounting to 0.5–2% in mass, hinting at a similar concentration on the oceanic layer [54]. Since liquid water from the deep interior is actively erupting into space, studying the interior is simpler and makes the satellite particularly appealing [64]. Using very low-resolution mass spectrometers to make in situ measurements of Enceladus’ plume, the Cassini Saturn Orbiter has shown that such sampling is feasible [64]. The Enceladus Life Finder (ELF) expands on previous achievements by analyzing the plume gas and particles using two high-resolution mass spectrometers and by using different tests for life [65].

Data from the ejected material showed three groups of solid particles being expelled: (1) essentially pure water ice, (2) water ice together with some macromolecular organic component, and (3) water rich in ions like Na^+^, Cl^−^, ${\mathrm{CO}}_{3}^{2-}$, and K^+^. The presence of macromolecules suggests the need for a layer of insoluble organics on top of the ocean, similar to some places on Earth [54,66]. Regarding the salts, these are evidence of the interaction between the ocean water and the rocky layer providing minerals to the ocean [66]. On a flyby through Enceladus’ plumes, the Cassini spacecraft also found levels of some molecular species like hydrogen, which is only justified by a continuous supply of this gas [64]. This suggests ongoing hydrothermal activity like serpentinization [64], which produces heat and might contribute to the thermal evolution of small bodies [67]. The hydrogen release is relevant since it can then be used by putative microbial life, via hydrogenotrophic methanogenesis to produce methane [64].

Regarding the organic matter, analyzing the carbon isotope ratios of the detected methane, as well as the pattern of carbon isotopes in non-methane hydrocarbons (ethane, ethylene, and propane), is the most effective way to discern between biogenic and abiogenic CH_4_ on Earth [68]. For instance, there would be a definite difference in the carbon isotopes between the methane and the carbon dioxide if methanogens produced the former from the latter. With laboratory samples, it would be simple to measure these isotopic alterations [68]. However, it is more problematic to design such equipment for a spacecraft, and the Cassini orbiter was not equipped to do so [68].

Nonetheless, an important result of Cassini’s ten-year exploration of this active world was the discovery of the intricate structure and dynamics of the Enceladus plume [69]. This discovery provides a compelling basis for future spacecraft exploration and studies on Earth to clarify the genesis and dynamics of the Enceladus geysers and their physical and chemical relationship to the subsurface ocean [69].

It is conceivable that life could exist in both the oceans of Enceladus and Europa based on the supposition that hydrothermal vent conditions comparable to those on Earth, where life can emerge, also exist in these moons [70]. Furthermore, it might be possible to find biosignatures in plume samples, depending on how much microbial life exists in the water.

Further research and observation of Enceladus’ ejected material requires remote sensing from Earth because a new expedition to Saturn is unlikely until 2030. However, remote sensing from the Earth to Enceladus provides much less information than in the case of Europa. Since many of the interesting organic chemical entities have transitions bellow the wavelengths of a millimetre, spectroscopy is a good tool for such enquiries [68]. While single-dish telescopes are useful for temporal monitoring, one drawback is that they cannot trace regions as vast as the plumes. Drabek-Maunder et al. [68] studied the concentration of a possible biomarker, methanol, by these means [68]. The possibility that the observed methanol is being created by living organisms within Enceladus’ subsurface ocean is interesting because if we assume that the methanol production within the subsurface ocean on Enceladus is similar to Earth, and if all of the carbon measured in Enceladus’ vents is expelled by microbes in the subsurface ocean, then we would expect a CH_3_OH:H_2_O abundance within Enceladus’ subsurface oceans to be around 0.015% in an organic environment, which is similar to the CH_3_OH abundance measured in the direct vicinity of the vents by Cassini at around 0.01% [68].

## 3. Planetary Field Analogues of the Icy Moons

For astrobiologists to assess the potential for habitability on extraterrestrial bodies, they rely on terrestrial environments with comparable conditions to those found on the planetary body of interest [71]. These are called planetary field analogues and they refer to places on Earth with extreme conditions, i.e., sites experiencing physical stress, such as high and low temperatures, pressures, and radiation doses, as well as chemical stress such as high mineral content, high and low pH, and low water availability [71]. Such environments on Earth may mimic the acidic clouds of Venus, the cold, dry plains and caves of Mars, and the icy moons of Jupiter and Saturn [18,72]. When considering extreme environments on Earth experiencing cold temperatures, the two polar regions are high contestants [22]. To meet the interconnected challenges of exploring Earth’s abyssal regions, and the subsurface oceans of icy moons as potentially analogous environments, it is becoming increasingly relevant to characterize potential synergies and exchangeable technologies within robotic engineering, mission autonomy, and sensor integration [73]. For both exploratory scenarios, the identification of abiotic biomimetic structures and the development of power storage and mobility generation systems are important scientific and technological endeavours [73]. For the exploration of extra-terrestrial ocean worlds, robotic designs with characteristics of conventional underwater vehicles (such as autonomous underwater vehicles–AUVs–and rovers) are being considered. Exo-oceanic bodies can be represented by various strata on our planet, e.g., deep sea and the ice caps with their interior lakes [73]. Analysis of planetary field analogues also allows the development and assessment of detection methods of biosignatures, including the establishment of baseline abiotic signatures, the evaluation of biosignature preservation, and the identification of chemical, physical, and isotopic traces left by terrestrial biology in conditions similar to icy moons [22].

The polar regions of Earth hold several unique characteristics useful for the research of habitability conditions in icy moons [22]. On a broad scope, both Antarctica and the Arctic at most locations are very dry, cold, and heavily irradiated [74]. Moreover, some sites encapsulate very complex systems of inorganic chemistry events, extremophiles ecology, and extra-terrestrial-like landscapes [74]. All this considered, polar regions are frequently used as planetary field analogues of icy worlds, and a couple of examples will now be detailed.

Analogue studies in the Canadian Arctic are abundant [75], for instance, in the Axel Heiberg Island, mainly on the ancient, large-scale evaporites formed by the interaction of salts with permafrost, which resemble springs that emerge from a single vent [75]. Seven perennial springs discharging water with high concentrations of salts are important due to their resemblance to the volcanic activity on Europa [75]. This site proves useful to understand local geophysics and extrapolate to the tectonic processes within the ice shell of icy moons and how ocean fluids are incorporated into it [75]. In order to study, for instance, Europa’s sulphur-rich surface, Ellesmere Island in the Canadian High Arctic may be sampled. This region of the North Pole may be the best-known chemical analogue and offers a rare chance to study sulphur minerals in combination with ice in a terrestrial setting [75]. Note that the best analogues are where ongoing geothermal activity is present together with ice. Sulphur versatility in metabolic processes and its abundance in Europa’s surface, makes this element very interesting for astrobiological studies [67]. Furthermore, sulphates present a spectroscopic absorbance dependent on the composition and temperature of the environment [72]. Autonomous detection techniques were used on samples from Ellesmere Island to track the formation and size of salt deposits, which exhibit spectrum characteristics resembling low temperature bound-water absorptions in Europa’s non-ice material [72].

## 4. Spectroscopy of Biosignatures Present on Ice

### 4.1. Biosignatures

A biosignature can be defined as the physical or chemical result of the actions of biology [76]. They can be direct or indirect results of metabolism, spectral characteristic on the reflection or scattering of light by organisms, or variations resulting from actions expected within the biosphere [77]. The discovery of a biosignature on a distant icy moon must be critically assessed [77]. Some concentration of CH_4_ might be biogenic but it could just as well be a result of serpentinization [78]. The amount of analyte being detected, together with the duration of such detection, might be enough to rule a biosignature as still misunderstood biotic chemistry instead of a remnant of life [77]. Life can alter the spectral fingerprint of a planet through a variety of different mechanisms [77]. The absorption and reflection of light by pigments, the scattering by the physical structures of individual organisms and community architectures, degradation products of biological molecules, fluorescence of pigments, and bioluminescence; all can affect the spectral imaging of a target object [77]. These examples, while being strong biosignatures can also have abiotic mimics [77]. Pigmentation has been developed by organisms for different purposes beyond light capture, carbon fixation, or metabolic energy demands. Some pigments have functions that include the screening of potentially damaging UV radiation, the quenching of free radicals, and protection against extreme temperatures [79]. Signatures related to these pigments could also serve as practical surface biosignatures [77]. Some microorganisms produce pigmentation in dark conditions meaning that their colouration has no relation to its function [77]. Starting from this observation, Schwieterman et al. [77] suggested that non-photosynthetic pigments can be used as alternative surface biosignatures. These scientists also prepared a classification of the molecular nature and spectral features of light harvesting pigments [77].

### 4.2. Spectroscopy of Biosignatures on Icy Planetary Field Analogues

Putative biosignatures present in icy moons may be detected by different spectroscopy techniques [23]. In order to prepare for upcoming life-detection space missions on icy worlds (see Section 4.3), it is necessary to perform analysis on planetary field analogues [23]. Using ice and water from Arctic samples, Coelho and co-authors [23] measured the reflection spectra of pigments that were isolated from 80 microorganisms. The results showed that dried samples had very high reflectance compared to wet samples, which suggests that signatures of surface biota could be more intense on icy moons, which are drier than Earth [23].

Complementarily, the Unidad Asociada UVA-CSIC at the Centro de Astrobiología in Spain created a remote Raman portable instrument that permitted spectral analysis in situ under cold circumstances, such as those encountered in the Arctic [80]. The device has demonstrated its ability to provide accurate and trustworthy spectral information on natural icebergs (the three internal vibrations of water molecules as well as their external modes, namely the broad band corresponding to the hydroxyl group stretching vibration (ν = 3000–3800 cm^−1^) up to 120 m away [80]. As a result, the remote Raman system could be a potent tool for in situ structural investigation of large ice surfaces, especially in situations when it is extremely challenging to sample the area or employ contact instruments [80].

Promising findings have also been obtained using UV spectroscopy to measure ambient light levels within ice cores [81]. NASA’s Jet Propulsion Laboratory together with the University of Southern California, the Montana State University, and Honeybee Robotics are working on The Wireline Analysis Tool for Subsurface Observation of Northern ice sheets (WATSON), an instrument for UV fluorescence analysis [81], capable of detecting the large variety of chemical molecules with astrobiological relevance whose absorption spectra fall within the 270–440 nm range, e.g., aromatic amino acids, single- and multi-ring aromatic hydrocarbons, and aromatic heterocycles [81].

With the improvement of technology, a better understanding of how to detect, identify, and characterize life and life-related chemicals that may exist or have existed on icy moons can be accomplished [81]. For that, the detection of biosignatures in harsh conditions like the polar regions must be practised and enhanced [81].

Resorting to spectroscopy analyses of space analogues is not rare [82]. Martian chemistry and dynamics are vastly analyzed through samples of similar conditions on Earth [82]. Raman and IR analysis for analogues of icy moons have also been reported, but employing UV-Vis for the same objective (i.e., search of biosignatures on ice samples as proxy for icy moons) is a growing approach [82]. This latter technique is fully justified due to its capability of unmixing complex spectra, relative accessibility, and an ability to be performed in extreme environments [82].

### 4.3. Spectroscopic Observations and Analyses on Space Missions to Icy Moons

Much has been learned about the chemistry of the surfaces of planets due to remote sensing in the ultraviolet and infrared regions of the electromagnetic spectrum [83]. Numerous surface elements, including rock-forming minerals, water ice, unusual volatiles, and organic molecules, have been found by telescopic studies as well as, in more recent years, spacecraft mission observations [83]. We can constrain internal processes like cryo-volcanism and aqueous geochemistry by identifying surface components and charting their distributions [84].

In most cases, spectra of known materials collected under controlled circumstances are used to compare spectral measurements of planetary surfaces [83]. An exact match implies that the composition, structure, and physical circumstances have been replicated. However, there is always the risk that some other material may give a similar signal, hence spectral interpretations are still vulnerable to the question of uniqueness [83]. Most satellite spectrometers detect radiance with a sensor, using that measurement to calculate surface reflectance [83]. Thus, it is necessary to consider atmospheric transmission and absorption if there is an atmosphere [83]. Mission observations are often made of reflected solar UV, visible, and infrared radiation [83].

The surface components, textures, and temperatures of the icy moons of the giant planets were all extensively studied [84]. Table 1, Table 2 and Table 3 summarize the equipment of the space missions sent to these frozen worlds, as well as future space missions. The Voyager I and II, Galileo, and Cassini missions were all equipped with imaging instruments and were designed with spectrometers covering the visible spectra but also capable of detecting ultraviolet and near-infrared radiation [83]. In addition, Voyager and Galileo had photopolarimeters systems to observe UV, visible, and IR radiation, whereas Cassini had a thermal infrared system [83]. Sensitive charge-coupled device (CCD)-based equipment onboard Galileo and Cassini’s satellites have supplied crucial information on both the surface compositions and distributions of surface components of icy Galilean and Saturnian moons [83]. Similar instruments on future missions, such as those envisaged for the exploration of icy moons orbiting Jupiter or Saturn, are likely to have even higher spectral resolution and coverage [83].

It has been suggested that a wide variety of compounds make up the ice satellite surfaces in the outer solar system [84]. Researchers will be able to look for these materials in spectrum measurements from previous, present, and upcoming spacecraft missions as a result of experimental spectroscopy of these compounds under well-monitored settings [83]. Although observations of complex biosignatures on the surface of icy moons are scarce, much has been achieved towards the detection of smaller molecular structures, which will help interpret future in situ spectroscopic analyses of molecules of astrobiological interest. The presence of hydrated chlorinated species on the icy crust of Europa may reveal information on the make-up of underground liquid water reservoirs, aiding in the assessment of its potential habitability [51]. Spectral unmixing modelling performed on telescopic observations in the NIR region on the ESO/Very Large Telescope, hypothesized that chlorinated species exist on the surface of Europa [97]. Hydrated magnesium chloride offers an excellent fit for the spectral absorption bands with peaks at 1.73 and 2.07 μm, however, epsomite (MgSO_4_·7H_2_O) would also appear on the band at 2.07 μm [97]. Although the trailing hemisphere also experiences radiolytic sulphur modification, these Mg-chlorinated compounds appear to be associated on a broad regional scale with geomorphologic features such as chaos terrains and dark regions [98].

In order to offer a solid experimental basis helpful for the interpretation of new remote sensing data obtained from both ground and space-based observations, it is crucial to conduct laboratory investigations of chlorinated species. Previous work by Thomas et al. [99], Henley et al. [100] and Bishop et al. [101,102] only assessed the reflectance spectra of chlorinated compounds at either NIR wavelengths or room temperature. However, the study by Angelis et al. [51], where magnesium chlorides were tested for their reflectance spectra in the 0.5–4.7 μm range in three different grain sizes, describes the spectral behaviour of those salts in sub-zero conditions. These types of experiments are beneficial for understanding data that will be returned by planetary missions [51].

In addition to the salts, the discovery of ammonium minerals on the exterior of planetary bodies may offer crucial information on the communication between the interior (oceans/brines) and exterior (surface) of these planets, indicating a fresh and replenished surface [103]. The interpretation of NH_4_^+^ minerals from remote sensing data is not always simple because of their phases, just like on Earth [103]. The near-infrared spectra of ammonium salts with distinct grain sizes at cryogenic temperature were also measured. Studies show how the NH_4_^+^ ion’s five distinctive bands respond to low temperature. Their spectra exhibit significant similarities as they cool: the band parameters, e.g., area and depth, typically increase with decreasing temperature but also some spectral features become more distinct. If ammonium-bearing minerals are present, they should be detectable by remote sensing due to distinctive spectral characteristics in the VIS and NIR regions [103].

Regarding carbonates, with the Galileo Mission, it was discovered that the spectrum characteristics of these species closely resembled those seen by the near-infrared mapping spectrometer (NIMS) [104]. By using cryogenic laboratory reflectance spectroscopic techniques, it has been possible to distinguish the compositional signatures of exogenic magnetospheric charged particles from those caused only by endogenic materials [104]. Abundances obtained from NIMS remote sensing may be able to shed light on the chemical reaction pathways that are taking place at the surface [104].

With the information from Galileo NIMS, a spectral library for salts at different temperatures in Europa has been developed [105,106,107], showing the spectra of sulphuric acid hydrate (H_2_SO_4_·nH_2_O), magnesium sulphate undecahydrate (meridianiite, MgSO_4_·11H_2_O), epsomite (MgSO_4_·7H_2_O), hexahydrite (MgSO_4_·6H_2_O), mirabilite (Na_2_SO_4_·10H_2_O), bloedite (Na_2_Mg(SO_4_)_2_·4H_2_O), and frozen sodium and magnesium sulphate brines.

The presence of oxygen in the surface bound exosphere, believed to have been created by violent particle bombardment of the surface, as well as the abundance of H_2_O_2_, show that radiolysis dominates the surface chemistry on Europa [12]. The optically detected surface layer can undergo complete modification in a few decades at the expected energy inflow rates [12]. Europa’s 210 to 300 nm reflectance can shift due to temporal variations in the Jovian magnetospheric energetic plasma; these variations have been seen in Europa’s UV reflectance. Because CO and SO are substances that could potentially result from radiolysis, it is important to investigate related substances like H_2_SO_4_ and different carbon compounds [12]. Radiolysis effects must be considered when making predictions, characterizing, and identifying surface species on Europa [12]. Other molecules such as SO_2_ (being carbonates which are also candidates) have been suggested as the source of infrared fingerprints from Europa [107]. In addition, on the cold Galilean satellites, carbon dioxide has been detected [107]. This ubiquitous molecule may have originated as a radiolytic and photolytic by-product of carbonaceous material in water ice, but this is uncertain [12].

## 5. Conclusions

The Universe is vast and complex, rich in celestial bodies potentially capable of harbouring life. As long as the three pillars of habitability (i.e., chemical elements necessary for life, a source of energy, and liquid water) are met, life may potentially thrive. In the solar system, the subsurface water oceans of the icy moons Europa and Enceladus of the Jovian and Saturnian system, respectively, are strong candidates for the attention of astrobiologists. The hydrothermal vents that may be present on the silicate layer in contact with the liquid water strata may provide the organic ingredients for prebiotic chemistry. Currently, only the surface has been studied in Europa. While this is a major limitation to the understanding of the chemistry happening in the subsurface ocean, the frozen shell also holds valuable information due to resurfacing events. Regarding Enceladus, scientists have been able to assess some of the content of its ocean with the Cassini mission that collected material being ejected from the gigantic plumes in the south pole of this moon.

When future space missions, such as Europa Clipper and JUICE analyze the habitability conditions of ocean worlds, they will have to pay special attention to avoiding contamination and therefore to planetary protection. Recently, Smith and Hendrickson [108] described the main points of the NASA-JPL lead workshop entitled ‘Europa Clipper Planetary Protection Workshop’, in which three objectives were mentioned: (1) validate the probability of contamination modelling for Europa Clipper planetary protections; (2) validate the probability values of the contamination model, and (3) develop cooperation regarding future planetary protection priorities and research plans [108].

In order to support future space missions, scientists rely on analogue samples to test and develop protocols to be applied on planetary bodies. For the study of the icy moons, polar regions on Earth, so called planetary field analogues, have proven to be essential. When physical and chemical conditions in both the Arctic and Antarctica partly mimic conditions on and inside Europa and Enceladus, astrobiologists take that to advantage and perform fieldwork, the results of which are extrapolated for the extra-terrestrial counterpart. Such studies may include the search for biosignatures, i.e., any element, molecule, or feature that may be used as evidence for past or present life. Protocols that are successfully applied in the polar regions should be considered potential techniques to equip in space missions, e.g., spectroscopic analysis of biosignatures. UV-Vis spectroscopy is a very useful analytical tool for characterization of biological molecules and has been successfully applied in extreme environments on Earth. After appropriate development and testing, the possibility of including this methodology in the payload of future space missions, which will perform in situ measurements, could provide key information about one of humankind’s most ancient questions: Are we alone in the Universe?

## Figures and Tables

**Table 1:** Missions to the icy moons of Saturn equipment for spectroscopy analysis of icy moons onboard.

Icy Moons of Saturn					
Mission	Year	Instrument	Spectral Range	Instrument Finding	References
Cassini	1997	Image Science Subsystem (ISS)	200–1100 nm	Images revealed a plume of tiny ice particles emerging from Enceladus’ south pole. The plume was identified in November 2005 as discrete jets, or geysers, carried upward by water vapour. These jets are responsible for ejecting the particles that will eventually supply Saturn’s E ring.	[85,86]
		Visible and Infrared Mapping Spectrometer (VIMS)	350–5100 nm	The majority of the particles in Enceladus’ plumes are made up of fine-grained water ice, ejected from the planet at speeds of 80–160 m/s and only a small percentage of these particles can escape from Enceladus. A stratigraphic link between tectonic structures and cryovolcanic activity is suggested by the correlation of particle sizes with geologic features and surface ages on Enceladus.	[87,88]
		Composite InfraRed Spectrometer (CIRS)	10–1400 cm^−1^	Endogenic thermal emission at temperatures as high as 190 K from the Enceladus tiger stripes, with significant spatial variation on scales ranging from tens of kilometres to tens of metres.	[89]
		Ultraviolet Imaging Spectrograph (UVIS)	EUV 563–1182 Å/FUV 1115–1912 Å	Mapped the structure of Enceladus’ and determined that its irregular eruptions primarily release water vapour. Over the length of the mission, there were no noticeable fluctuations in the volume of water vapour, while the largest jets, which carry most frozen grains, vary more.	[90]

**Table 2:** Mission to the icy moons of Jupiter and equipment for spectroscopy analysis of icy moons onboard.

Icy Moons of Jupiter					
Mission	Year	Instrument	Spectral Range	Instrument Finding	References
Galileo	1981	Solid State Imager (SSI)	375–1100 nm	Provided evidence of geologic activity on Europa that backs up the theory that there is liquid water underneath a thin layer of ice.	[91,92]
		Near-Infrared Mapping Spectrometer (NIMS)	700–5200 nm	Improved the understanding of the composition of Europa’s ice shell.	[93]
		UltraViolet Spectrometer (UVS)	113–432 nm	Examined the Galilean satellites’ surface composition and escape of volatile molecules.	[94]
		Extreme Ultraviolet Spectrometer (EUVS)	54–128 nm	Examined the Galilean satellites’ surface composition and escape of volatile molecules.	[94]
JUICE	(Planned to) 2023	Moons and Jupiter Imaging Spectrometer (MAJIS)	0.4 to 5.7 μm		[95]
		Jovis, Amorum ac Natorum Undique Scrutator (JANUS)	350–1050 nm		[95]
		Ultraviolet Spectrograph (UVS)	55–210 nm		[95]
Europa Clipper	(Planned to) 2024	Europa Imaging System (EIS)	350–1050 nm		[96]
		Europa Thermal Emission Imaging System (E-THEMIS)	7–50+ μm		[96]
		Europa Ultraviolet Spectrograph (Europa-UVS)	55–206 nm		[96]
		Mapping Imaging Spectrometer for Europa (MISE)	0.8–5 μm		[96]

**Table 3:** Missions to both gas giants and equipment for spectroscopy analysis of icy moons onboard.

Icy Moons of Jupiter and Saturn					
Mission	Year	Instrument	Spectral Range	Instrument Finding	References
Voyager I and II	1977	InfraRed Interferometer Spectrometer (IRIS)	180–2500 cm^−1^ 5000–30,000 cm^−1^	Analyzed the atmospheres of Jovian satellites, and their vertical temperature profiles.	[2]

## Data Availability

Not applicable.
